# Telephone Versus Internet Administration of Self-Report Measures of Social Anxiety, Depressive Symptoms, and Insomnia: Psychometric Evaluation of a Method to Reduce the Impact of Missing Data

**DOI:** 10.2196/jmir.2818

**Published:** 2013-10-18

**Authors:** Erik Hedman, Brjánn Ljótsson, Kerstin Blom, Samir El Alaoui, Martin Kraepelien, Christian Rück, Gerhard Andersson, Cecilia Svanborg, Nils Lindefors, Viktor Kaldo

**Affiliations:** ^1^Osher Center for Integrative MedicineDepartment of Clinical NeuroscienceKarolinska InstitutetStockholmSweden; ^2^Division of PsychologyDepartment of Clinical NeuroscienceKarolinska InstitutetStockholmSweden; ^3^Division of PsychiatryDepartment of Clinical NeuroscienceKarolinska InstitutetStockholmSweden; ^4^Department of Behavioural Sciences and LearningLinköping UniversityLinköpingSweden

**Keywords:** Internet, telephone, self-report measures, missing data, method validation

## Abstract

**Background:**

Internet-administered self-report measures of social anxiety, depressive symptoms, and sleep difficulties are widely used in clinical trials and in clinical routine care, but data loss is a common problem that could render skewed estimates of symptom levels and treatment effects. One way of reducing the negative impact of missing data could be to use telephone administration of self-report measures as a means to complete the data missing from the online data collection.

**Objective:**

The aim of the study was to compare the convergence of telephone and Internet administration of self-report measures of social anxiety, depressive symptoms, and sleep difficulties.

**Methods:**

The Liebowitz Social Anxiety Scale-Self-Report (LSAS-SR), Montgomery-Åsberg Depression Rating Scale-Self-Rated (MADRS-S), and the Insomnia Severity Index (ISI) were administered over the telephone and via the Internet to a clinical sample (N=82) of psychiatric patients at a clinic specializing in Internet-delivered treatment. Shortened versions of the LSAS-SR and the ISI were used when administered via telephone.

**Results:**

As predicted, the results showed that the estimates produced by the two administration formats were highly correlated (*r*=.82-.91; *P*<.001) and internal consistencies were high in both administration formats (telephone: Cronbach alpha=.76-.86 and Internet: Cronbach alpha=.79-.93). The correlation coefficients were similar across questionnaires and the shorter versions of the questionnaires used in the telephone administration of the LSAS-SR and ISI performed in general equally well compared to when the full scale was used, as was the case with the MADRS-S.

**Conclusions:**

Telephone administration of self-report questionnaires is a valid method that can be used to reduce data loss in routine psychiatric practice as well as in clinical trials, thereby contributing to more accurate symptom estimates.

## Introduction

Self-report measures are widely used in both routine psychiatric care and in clinical trials as they have several advantages including psychometric properties similar to clinician-administered instruments [1], low cost, and the potential to administer the instruments over the Internet [2,3]. However, a common problem in these settings is data loss. As pointed out by Claassen et al [4], even in randomized controlled trials attrition rates can be 30-40% and in effectiveness studies on regular care patients this number is likely to be even higher. In an effectiveness study on Internet-based cognitive behavior therapy (ICBT) for panic disorder, we found that as few as 32% of patients completed self-report assessments at six-month follow-up, despite several text message reminders [5]. Data loss lowers the statistical power and as attrition could be non-randomly distributed (eg, persons with more severe symptoms may be more likely not to fill out self-assessments), this could render skewed estimates of symptom levels and treatment effects [6]. A common way of handling this problem is through statistical procedures such as multiple imputation or the use of full-information maximum likelihood estimation models [7]. These missing data strategies do however have some important disadvantages, including non-testable assumptions of the randomness of the missing data patterns, difficulty of dealing with non-normally distributed covariates, and the complexity of the computational process [8].

One way of reducing the negative impact of missing data without the disadvantages of advanced imputational methods could be to increase completion rates of self-reports through the use of telephone assessments, that is, telephone administration of self-report measures as a means to complete the data missing from the online data collection. Several studies have demonstrated that diagnostic assessment interviews can be conducted over the telephone with high convergent validity with face-to-face interviews [9,10]. However, a diagnostic interview or a clinician’s expert rating of the patient’s level of symptoms is not equivalent to a telephone-administered self-report, where the clinician’s impact on the ratings is put to a minimum by using standardized questions and answers that are read verbatim to the patient. The body of evidence is limited when it comes to how accurate this kind of telephone administration of self-report instruments is in comparison to the standard way of self-assessment. We have found only two studies investigating this. In these studies, it was shown that the Penn-State Worry Questionnaire (PSWQ), the Beck Depression Inventory (BDI), and the General Health Questionnaire (GHQ) could be completed over the telephone providing similar estimates as when administered as self-assessment using paper and pencil [11,12]. Another problem when providing self-assessments over the telephone with patients who have failed to complete standard self-assessments is that the patient’s motivation to devote a substantial amount of time for a telephone interview might be limited. This problem could be even more pronounced in long-term follow-ups. Against this background, it is important to use instruments with few items when conducting telephone-administered assessments with self-report measures.

To our knowledge, no prior study has investigated whether telephone and Internet administration of self-report measures produce equivalent results in the assessment of social anxiety, depressive symptoms, and sleep difficulties. More knowledge in this regard could lead to more effective strategies for handling data loss in clinical routine psychiatric care as well as in clinical trials. Also, investigation of whether it is possible to use shortened, and thus more efficient, versions of the full-length scales over the telephone has to our knowledge not been done.

The main aim of this study was to compare the convergence of telephone and Internet administration of self-report measures of social anxiety, depressive symptoms, and sleep difficulties. The Liebowitz Social Anxiety Scale - Self-Report (LSAS-SR) [1], Montgomery Åsberg Depression Rating Scale - Self-Rated (MADRS-S) [13], and the Insomnia Severity Index (ISI) [14] were used. We hypothesized that the estimates produced by the two administration formats would be highly correlated.

As a secondary aim, we wanted to explore three different strategies for developing an interview version of a self-report measure. The first is the most straightforward, as the same questions and response options are used in the interview as in the self-report measures. This was used when comparing Internet-administered MADRS-S to a telephone interview version of the same measure. The second strategy emerged from the need to keep the telephone interviews short. We explored this by reducing the number of items for the LSAS-SR and ISI when the measures were telephone-administered. Thus, we compared the Internet-administered full-scale self-report versions against shortened or full-scale telephone-administered versions of the same measure. The third strategy was to use a different measure within the same symptom domain in the telephone interview compared to when administering the measure via the Internet. The main reason to use this strategy is when the nature of the questions and answers in the self-report measure are deemed somewhat difficult to administer verbally over the telephone. This is the case with MADRS-S, which has long questions and answers are given on a 7-point scale with four anchor labels that are also quite long and unique for each question. Specifically, we investigated whether a shortened telephone-administered version of the Hospital Anxiety and Depression Scale (HADS) [15], deemed to be easier to administer via the telephone, could be as highly correlated with Internet-administered MADRS-S as the telephone-administered MADRS-S.

## Methods

### Design

This study employed a repeated measurement design where participants provided data in both administration formats, that is, telephone and Internet. Participants completed the Internet-administered self-report questionnaire first, followed by a telephone-administered assessment with the same questionnaire, shortened or full-scale. The average time between assessments was 3.1 days (SD 2.2) and the range was 0 to 7 days. The sample (N=82) comprised three cohorts: (1) participants seeking treatment for social anxiety disorder (SAD), denoted SAD sample (n=14), (2) participants seeking treatment for depression (DEP), denoted DEP sample (n=35), and (3) participants diagnosed with insomnia (Insomnia sample, n=33). Type of self-report measure used and whether the full version of the measure was telephone-administered were as follows: the SAD sample completed the full version of the LSAS-SR via the Internet and a short version of LSAS-SR via telephone; the DEP sample completed the full version of the MADRS-S via the Internet and the full version via telephone; and the Insomnia sample completed the full versions of the ISI and MADRS-S via Internet and short versions of the ISI and HADS via telephone.

### Recruitment and Participants

Participants were recruited from a series of patients seeking treatment at the Internet-based Cognitive Behavior Therapy Clinic (ICBT clinic) located at the Karolinska University Hospital Huddinge (Psychiatry Southwest) in Stockholm, Sweden. The ICBT clinic provides Internet-based CBT, which is a treatment that essentially can be described as guided online CBT-bibliotherapy with therapist contact through an Internet-based messaging system resembling email [16]. The ICBT clinic treatment context has been described previously in greater detail [5]. Participants were self-referred and could apply through the official website of the ICBT clinic. Only participants who completed the two assessments within one week (on the Internet and via telephone) were included in the present study. Table 1 presents a demographic description of the participants.

**Table 1:** Description of the participants.

		SAD^a^sample (n=14)	DEP^b^sample (n=35)	Insomnia sample (n=33)
**Age, mean (SD)**		31.7 (12.5)	36.5 (10.1)	47.2 (13.6)
**Gender**				
	Women (%)	9 (64.3)	21 (60.0)	24 (72.7)
	Men (%)	5 (35.7)	14 (40.0)	9 (27.3)
**Marital status**				
	Married or de facto (%)	7 (50.0)	21 (60.0)	21 (63.3)
	Not married (%)	7 (50.0)	14 (40.0)	12 (36.7)
**Parental status**				
	Parent	4 (28.6)	16 (45.7)	23 (69.7)
	Not parent	10 (71.4)	19 (54.3)	10 (30.3)
**Education**				
	Did not finish high school (%)	4 (28.6)	7 (20.0)	1 (3.0)
	Finished high school (%)	8 (57.1)	7 (20.0)	8 (24.2)
	University (%)	2 (14.3)	21 (60.0)	24 (72.7)

^a^SAD: social anxiety disorder

^b^DEP: depression

### Measures

#### Social Anxiety

The LSAS-SR was used to assess social anxiety. The LSAS-SR measures fear in and avoidance of 24 social situations (13 performance and 11 interaction situations) that are assumed to be difficult for people suffering from social anxiety disorder. The LSAS-SR is highly correlated with the clinician-administered Liebowitz Social Anxiety Scale (*r*=.85) [1]. LSAS-SR has high internal consistency (Cronbach alpha=.95), as well as high test-retest reliability over 12 weeks (*r*=.83) [17]. The convergent and discriminant validity of LSAS-SR has been shown to be strong and the scale is sensitive to change and is therefore often used in treatment research [17]. When administered via the telephone, a shortened version of the LSAS-SR was used. This short version was derived through factor analysis based on previously collected clinical data from patients with SAD at the ICBT clinic (N=684). Ten situations (rated for both fear and avoidance) were chosen for the short version, based on their correlations with the total scale score while ensuring that items from all factors that emerged in the principal components analysis were represented, in order to avoid making the short version narrower in measurement scope than the full version. The correlations with the full scale were *r*=.96 (total), *r*=.95 (fear), and *r*=.95 (avoidance). The included items, as numbered in the full scale, were 4, 9, 10, 11, 12, 15, 16, 17, 20, and 23.

#### Depressive Symptoms

We used the MADRS-S and a shortened version of the HADS to assess depressive symptoms. The full version of the MADRS-S was used partly because of its brevity in terms of number of items, partly as each of the items in the scale covers rather different dimensions of depressive symptoms, making item reduction difficult. MADRS-S consists of nine items measuring nine different symptoms and each symptom is rated on a 7-point scale with four predefined anchor labels and three non-defined anchor labels in between. The test-retest reliability of MADRS-S is high with *r* ranging from .80 to .94. In a comparative study, Svanborg and Åsberg [13] showed that MADRS-S correlated highly (*r*=.87) with BDI [18].

The HADS consists of two subscales: one that measures depressive symptoms and one that measures general anxiety. Each subscale has 7 items, each rated 0-3, yielding a total score between 0 and 42. The HADS has good convergent validity as the depressive symptoms subscale is highly correlated with the clinician-administered Montgomery-Åsberg Depression Rating Scale (*r*=.81) [15]. In a review of 71 studies investigating the psychometric properties of HADS, Bjelland et al [19] found that Cronbach alpha exceeded .60 in all of them, indicating stable and adequate internal consistency. In the present study, only a subset of items of the scale assessing depressive symptoms was used and this short version of HADS was solely telephone-administered and compared to the Internet-administered MADRS-S. We chose the four items of the HADS depression scale deemed most suitable for telephone-administration. These items, as numbered from the original scale, were 2, 4, 8, and 10.

#### Sleep Difficulties

The ISI was used to assess sleep difficulties. The ISI is a 7-item instrument assessing the severity of initial, middle, and late insomnia; sleep satisfaction; interference of insomnia with daytime functioning; noticeability of sleep problems by others; and distress about sleep difficulties. A 5-point scale (0-4) is used to rate each item, yielding a total score of 0 to 28. The ISI has adequate psychometric properties including high internal consistency (Cronbach alpha=.74) and is moderately correlated with other measures of sleep behaviors [14]. The items chosen for the shortened telephone version of ISI were items 1a, 1b, 1c, 2, and 3. These five items were chosen as they correspond to the DSM-IV diagnostic criteria of insomnia.

### Procedures

Participants completed Internet-administered assessments through the Internet-based platform of the ICBT clinic. Previous research has shown that the LSAS-SR, MADRS-S, HADS, and ISI can be administered via the Internet with psychometric properties equivalent to the paper-and-pencil versions [2,3,20]. Participants in the SAD sample filled out the LSAS-SR, while those in the DEP sample completed the MADRS-S, and the Insomnia sample completed the MADRS-S and the ISI. After this had been done, participants were contacted by a licensed psychologist or by a student at the master level psychology program who conducted the telephone assessment by reading the questions to the participant and recording the response. As described in the design, this meant that the SAD sample was administered a short version of the LSAS-SR, the DEP sample was administered the full version of the MADRS-S, and the Insomnia sample was administered both a short version of the HAD depression scale and a short version of the ISI. There were eight assessors in total and they followed a structured interview guide after having received education on how to conduct the telephone assessments. The interviewer first informed the respondent on how to give their answers, then read the instructions and questions of the respective instrument exactly as presented in the scale. For items with predefined anchor labels, the clinician read the corresponding text to the participant. Clinicians reading the self-report instrument to the participant were instructed not to make any form of independent assessment of the symptoms or to give any further explanation of how to interpret the question, but to only record the participant’s response.

### Statistical Analyses

Analyses were conducted using SPSS, version 20. Cronbach alpha was used to calculate internal consistency. Pearson’s zero-order product-moment correlation was used to analyze intercorrelations across administration formats. Data were standardized prior to correlation analyses by subtracting the mean score from each raw score and dividing by the standard deviation. To provide an estimate of how raw scores from the telephone-administered assessment translated into the full scale as completed online, linear regression analyses were conducted where Internet scores were regressed on telephone scores. *Z* tests were used to investigate differences in correlation coefficients between the measures.

## Results

### Internal Consistency

The alpha values for each questionnaire and administration format are presented in Table 2. Cronbach alpha ranged between .76 and .86 for telephone administration and .79 and .93 for Internet administration. The differences in internal consistency across administration format were small, with the largest difference being found for the LSAS-SR (telephone: Cronbach alpha=.86 vs Internet: Cronbach alpha=.93).

### Correlation Between Administration Formats

The scores from the telephone and Internet administered self-report questionnaires were all highly and significantly (*P*<.001 for all measures) correlated indicating strong positive associations of the two formats. The correlation coefficients were as follows: LSAS-SR (Internet) with short LSAS-SR (telephone), *r*=.82; ISI (Internet) with short ISI (telephone), *r*=.91; MADRS-S (Internet) with MADRS-S (telephone), *r*=.83; MADRS-S (Internet) with short HADS (telephone), *r*=.70. *Z* tests did not indicate any significant differences between correlations across questionnaires (*P*>.50).

### Regression Coefficients to Predict Internet Self-Report From Telephone Self-Report

In order to obtain an estimate of how the telephone-administered raw scores best translate into the Internet-administered version, regression coefficients were calculated for each measure where the Internet-administered scores were regressed on the telephone-administered scores. Table 3 presents the beta coefficients (ie, the change in the Internet-administered scales for a one-point increase in the telephone-administered scales), as well as the intercepts (ie, the score on the Internet-administered version when the telephone score equals zero). All beta coefficients were statistically significant indicating that the Internet-administered LSAS-SR can be predicted from the telephone-administered short version of LSAS-SR (*t*_1,12_=4.92, *P*<.001), the Internet-administered MADRS-S can be predicted from the telephone-administered MADRS-S (*t*_1,36_=8.76, *P*<.001) as well as from the short HADS depression scale (*t*_1,31_=5.43, *P*<.001), and finally, the Internet-administered ISI can be predicted from the telephone-administered ISI (*t*_1,31_=12.09, *P*<.001). Thus, missing Internet ratings can be estimated by using the general formula: Internet score = intercept + beta * telephone score.

**Table 2:** Internal consistencies (Cronbach alpha) for the two administration formats for each questionnaire.

Measure	Telephone administration^f^	Internet administration
LSAS-SR^a^	.86	.93
MADRS-S^b^	.76	.79
HADS^c,d^	.85	-
ISI^e^	.83	.87

^a^LSAS-SR: Liebowitz Social Anxiety Scale – Self-Report

^b^MADRS-S: Montgomery Åsberg Depression Rating Scale – Self-Rating

^c^HADS: Hospital Anxiety and Depression Scale

^d^HADS was only administered via telephone

^e^ISI: Insomnia Severity Index

^f^Shortened versions of the LSAS-SR, ISI, and HADS were used when administered on the telephone

**Table 3:** Mean, SD, and regression coefficients to predict Internet self-report from telephone self-report.

Measure	Administration format^f^		Regression coefficients, Internet data regressed on telephone data		
	Telephone, mean (SD)	Internet, mean (SD)	Intercept	Beta	*P* value of regression beta coefficient
LSAS-SR^a^	34.1 (9.6)	70.0 (20.0)	6.25	1.87	<.001
MADRS-S^b^	24.4 (7.2)	26.9 (7.0)	7.36	0.80	<.001
HADS^c,e^	2.6 (2.5)	-	5.77	2.88	<.001
ISI^d^	8.3 (4.7)	12.2 (5.9)	2.81	1.14	<.001

^a^LSAS-SR: Liebowitz Social Anxiety Scale - Self-Report

^b^MADRS-S: Montgomery Åsberg Depression Rating Scale - Self-Rated

^c^HADS: Hospital Anxiety and Depression Scale

^d^ISI: Insomnia Severity Index

^e^HADS was only administered via telephone and predicts MADRS-S in the regression results presented in the table

^f^Shortened versions of the LSAS-SR, ISI, and HADS were used when administered on the telephone

## Discussion

### Principal Findings

The aim of this study was to compare the convergence of telephone and Internet administration of self-report measures of social anxiety (LSAS-SR), depressive symptoms (MADRS-S), and sleep difficulties (ISI). As predicted, the results showed that the estimates produced by the two administration formats were highly correlated. The correlation coefficients were similar across questionnaires and the shorter versions of the questionnaires used in telephone administration of the LSAS-SR and ISI performed, in general, equally well compared to when the full scale was used, as was the case with the MADRS-S. The analysis also showed that a shortened telephone-administered version of a different scale assessing the same symptom domain could be used to predict Internet-administered self-report scores. In other words, shortened HADS could be used to predict the full MADRS-S with similar effectiveness as when the full telephone-administered MADRS-S was used to predict the Internet-administered MADRS-S. These findings suggest that providing self-report questionnaires over the telephone, in their full or shortened form, is a valid administration format for measures commonly used to assess social anxiety, depressive symptoms, and sleep difficulties.

As outlined in the introduction, prior research in this area is scarce and to our knowledge this is the first study to compare the psychometric properties of self-report measures administered via the telephone and the Internet. However, two prior studies have investigated the correlations between telephone and paper-and-pencil-administered self-report instruments. The present study has similar estimates on measures of association as in the study by Senior and colleagues [11] investigating the worry measure, PSWQ, and the depression inventory, BDI. Evans and colleagues [12] reported a correlation coefficient of .83 when comparing the GHQ administered over the telephone and as paper-and-pencil self-report, which is close to the estimates found in this study. This is further indication that Internet is a valid way of providing self-report questionnaires, which has also been previously demonstrated [2,21].

We regard the findings of the present study as relevant from a clinical as well as from a research perspective as they show that telephone administration can be a valid substitute for conventional use of self-report measures. As missing data is a substantial problem in both routine psychiatric practice and clinical trials, the findings of this study are important as they support the use of telephone interviewing of patients who have failed to provide self-report data. This, in turn, can lead to lower attrition rates and thereby more accurate estimates of symptom levels and treatment effects. As mentioned in the introduction, this type of handling of missing data has some advantages compared to using statistical procedures such as multiple imputation. A direct comparison between these forms of data replacements was beyond the scope of this paper, but should be investigated in future studies. One potential problem when trying to reach patients who have not completed self-report assessments is that their willingness to spend large amounts of time on the telephone being interviewed might be reduced. Therefore, a major implication of this study is that it is also possible to replace the full Internet-administered self-report version of the respective scales with shorter versions (LSAS-SR and ISI) or even with another set of questions within the same symptom category (HADS). This may further increase the possibility of reducing attrition rates, as not more than three or four minutes are required to complete the telephone assessments.

### Limitations

There were some limitations to this study. First and most importantly, there was no randomization of the order in which participants completed questionnaires. However, previous research has demonstrated limited effect of order [12]. Second, there was some time lag (a maximum of one week) between assessment points allowing for true natural fluctuations in symptom levels to occur. Considering that no treatment was initiated between the assessment points and that previous studies have found that social anxiety and depressive symptoms tend to be stable for this short period of time if untreated [22], this was nevertheless deemed as acceptable. It also reduced the risk of recall bias. Third, this study used a clinical sample, which may reduce the generalizability of the findings to non-clinical populations. It is however difficult to argue for a plausible mechanism for this potential difference and telephone assessment as data replacement method is probably most useful in clinical settings.

### Conclusions

In spite of these limitations, we regard the results of this study as important as they show that telephone administration of self-report measures of social anxiety, depressive symptoms, and sleep difficulties can be a valid method of administration. This procedure can be used to reduce data loss in routine psychiatric practice as well as in clinical trials, thereby contributing to more accurate symptom estimates.

## Abbreviations

BDI: Beck Depression Inventory
DEP: depression
GHQ: General Health Questionnaire
HADS: Hospital Anxiety and Depression Scale
ICBT: Internet-based cognitive behavior therapy
ISI: Insomnia Severity Index
LSAS-SR: Liebowitz Social Anxiety Scale - Self-Report
MADRS-S: Montgomery-Åsberg Depression Rating Scale - Self-Rated
PSWQ: Penn-State Worry Questionnaire
SAD: social anxiety disorder
